# Supplementary figures and images for: Fibroblast growth factor-9 expression in airway epithelial cells amplifies the type I interferon response and alters influenza A virus pathogenesis
Source: PLoS Pathog. 2022 Jun 8;18(6):e1010228. doi: 10.1371/journal.ppat.1010228 (PMC9212157; doi:10.1371/journal.ppat.1010228)

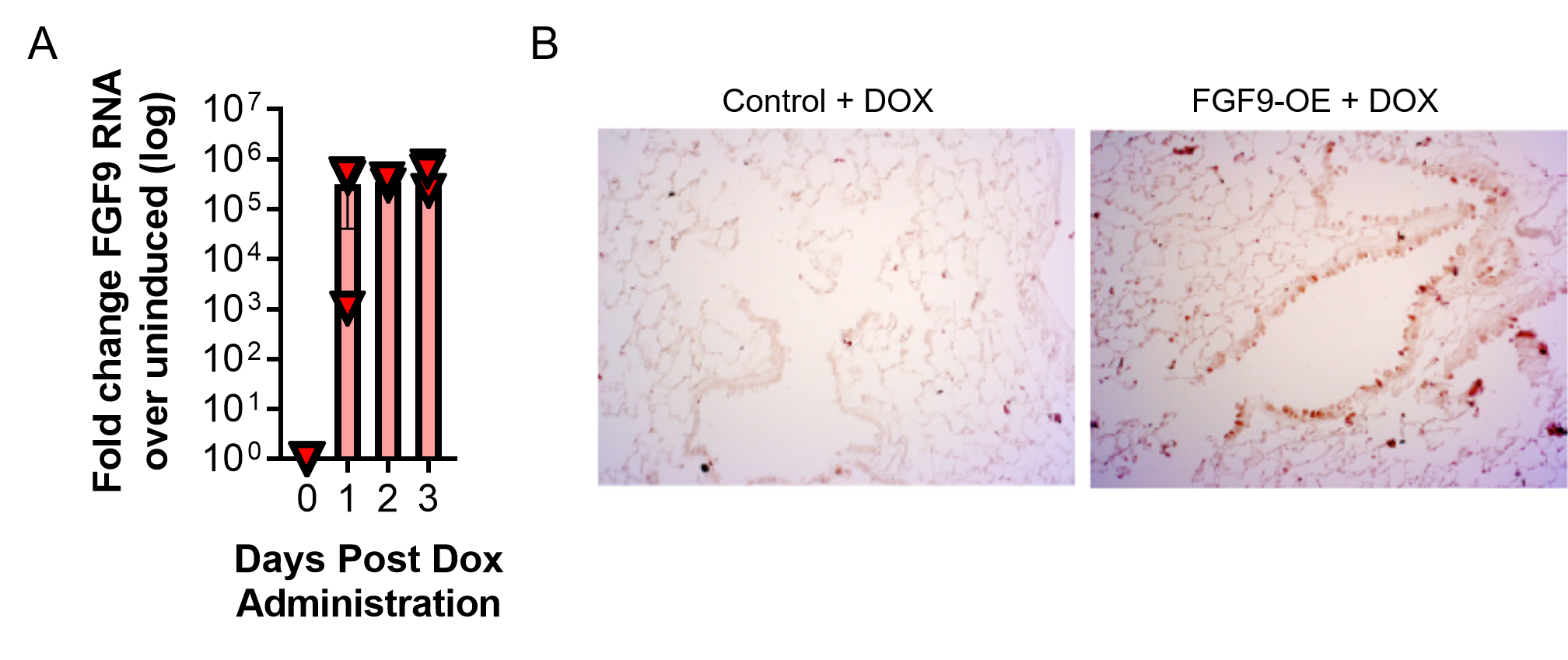

Supplement: S1 Fig — (A) DOX was administered to FGF9-OE mice for 1–3 days and Fgf9 was measured in whole lung RNA by RT-qPCR. Data are represented as fold change of Fgf9 compared to the uninduced lung (0 days post DOX administration). (B) FGF9-OE and control mice were given DOX for 3 days, lungs were harvested, and lung sections were analyzed for FGF9 expression by immunohistochemistry as described in Materials and Methods. (TIF) [file ppat.1010228.s001.tif]

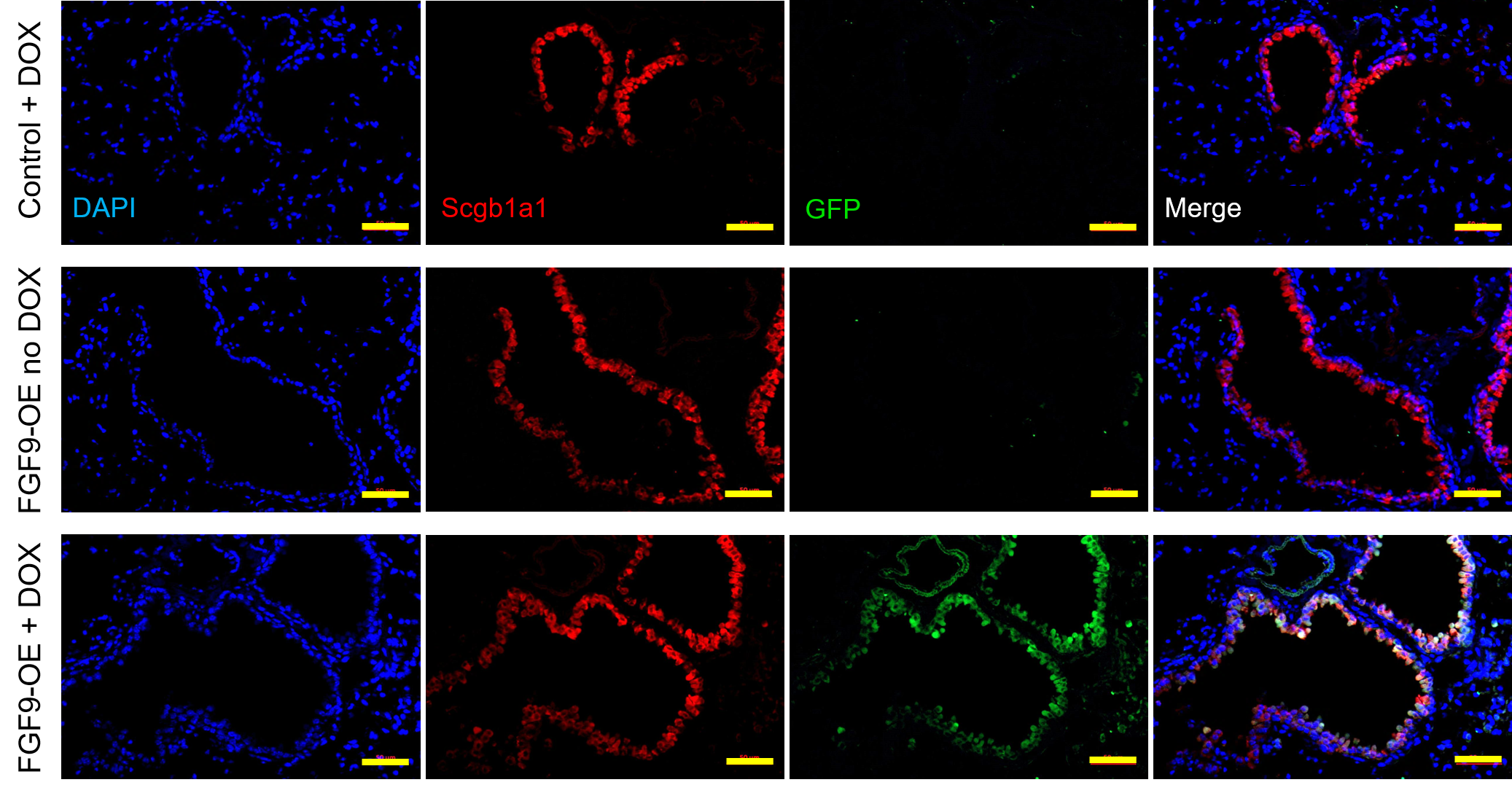

Supplement: S2 Fig — Representative images of lung sections from control mice or FGF9-OE mice administered DOX for 3 days and FGF9-OE mice without DOX, staining for DAPI (blue) and Scgb1a1 protein (red) and endogenous eGFP expression. Scale bars = 50 μm. (TIF) [file ppat.1010228.s002.tif]

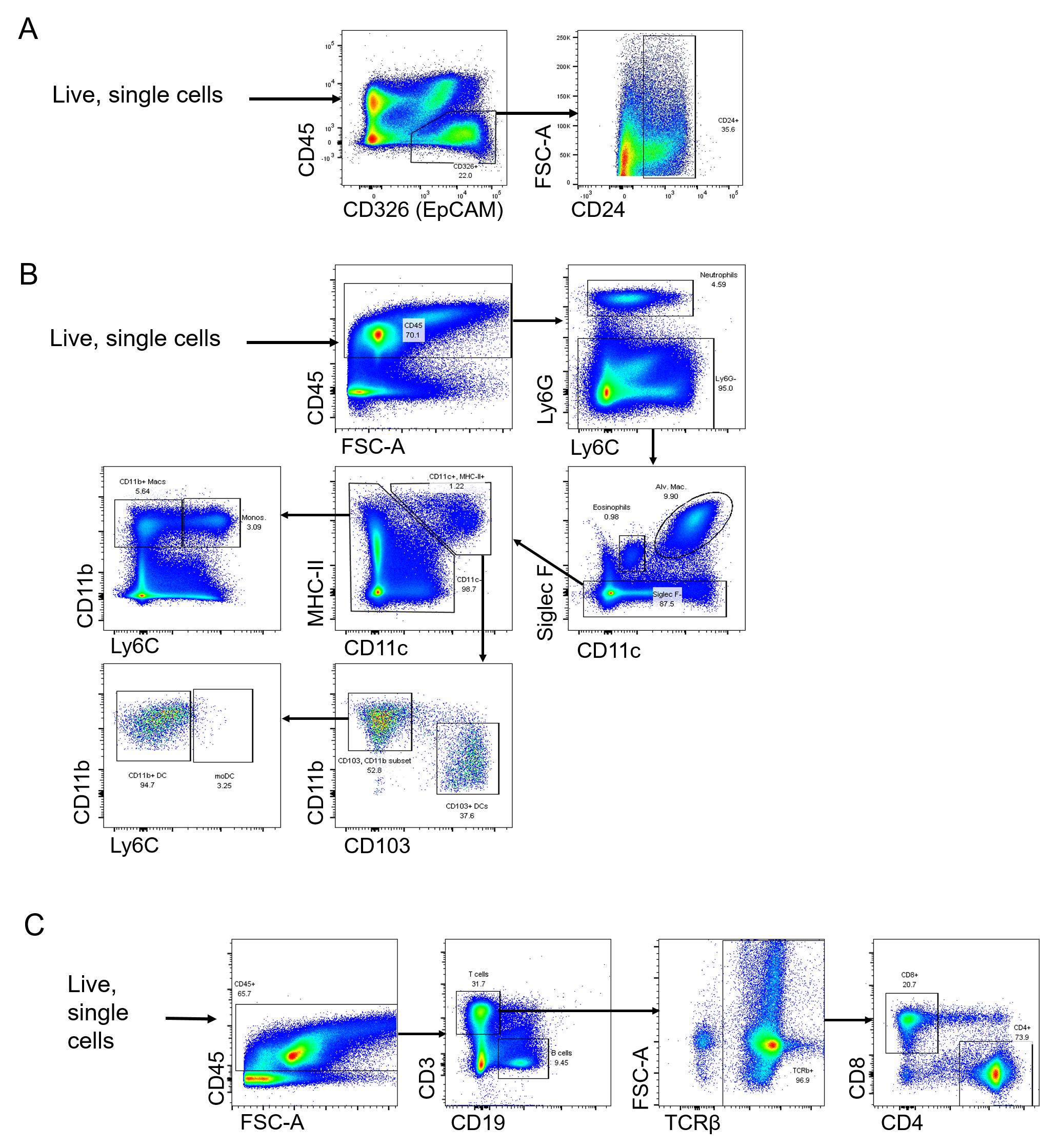

Supplement: S3 Fig — (A-B) Representative flow cytometry plots depicting the gating strategy for epithelial cell subpopulations (A) and innate and adaptive immune cell (B-C) subpopulations isolated from control mice lungs. (TIF) [file ppat.1010228.s003.tif]

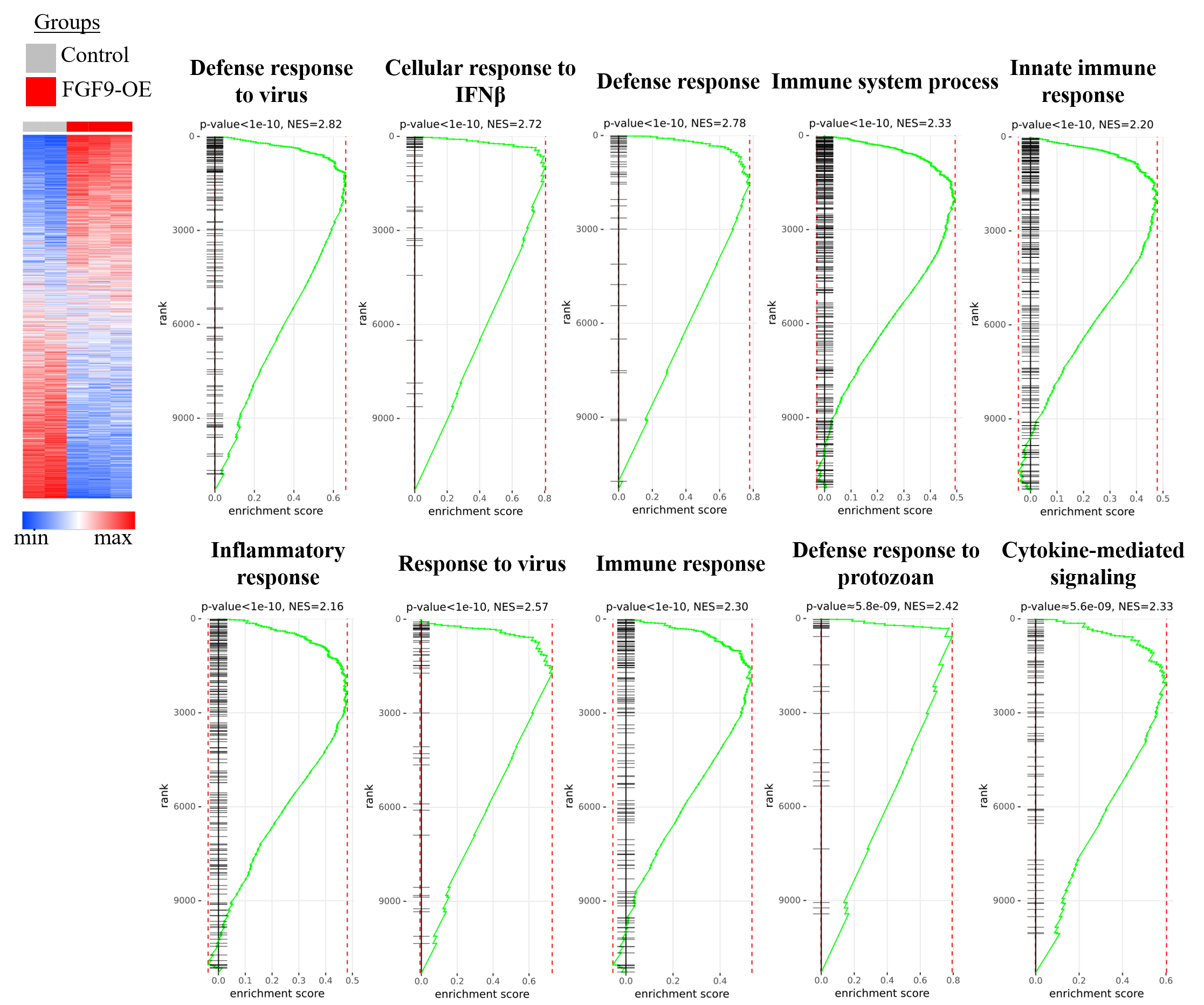

Supplement: S4 Fig — GSEA plots of the top ten most significant (by adjusted p-value) positively-enriched pathways found upregulated in the FGF9-OE airway epithelial cells as compared to control airway epithelial cells at 1 dpi. (TIF) [file ppat.1010228.s004.tif]

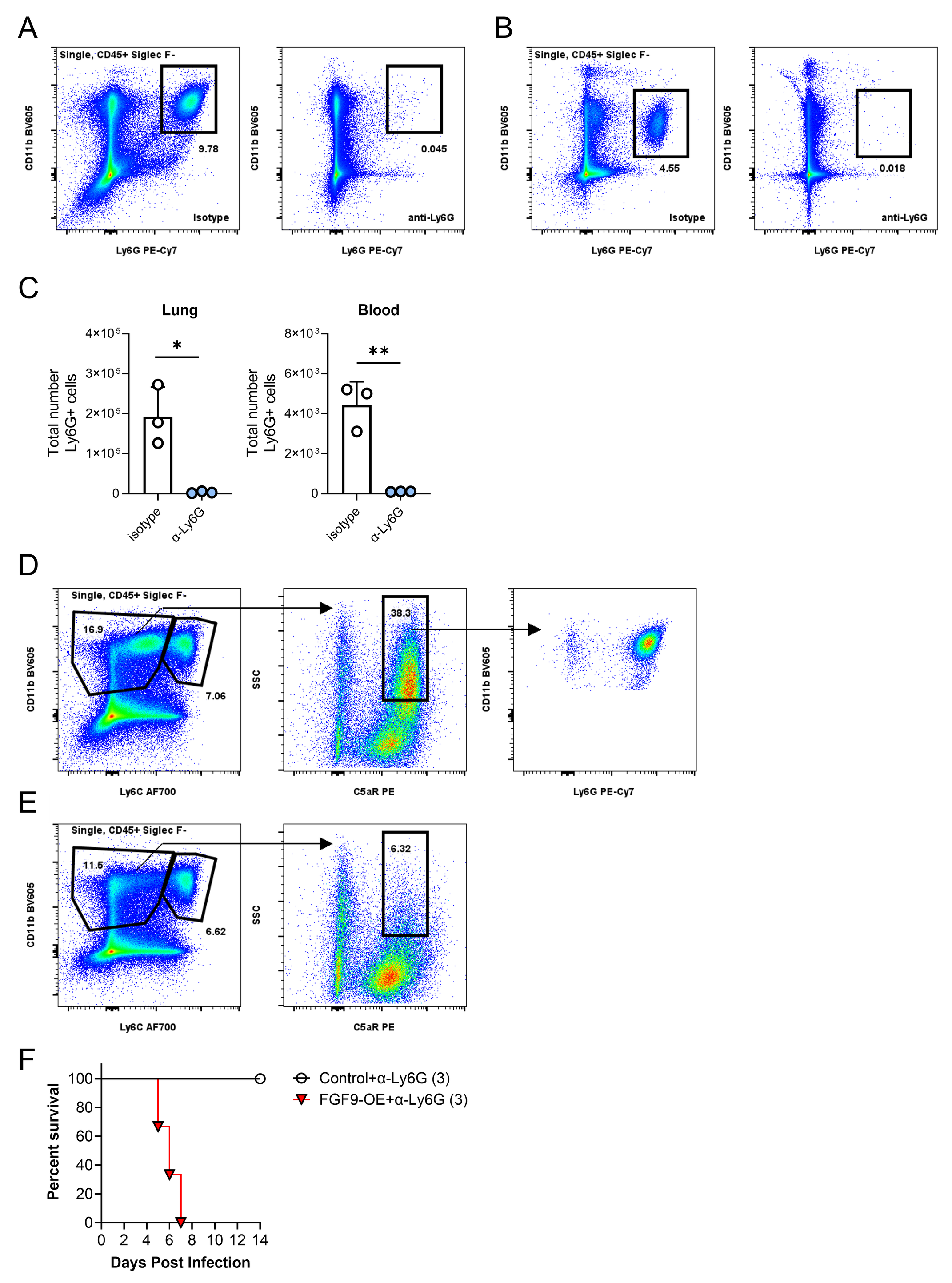

Supplement: S5 Fig — (A-E) WT FVB/NJ mice were given 0.25 mg anti-Ly6G or rat IgG2A isotype control antibody i.p. on d0 and concurrently infected with 6 x 10^4^ PFU IAV A/WSN/33 i.n. At 1 dpi, lungs and blood were harvested and analyzed for neutrophil depletion by flow cytometry. (A-C) Shown are representative flow cytometry plots for Ly6G+ CD11b+ expression on CD45+, Siglec F− singlets in the lungs (A) and blood (B) of isotype control-treated (left panel) mice and anti-Ly6G-treated (right panel) mice. Total Ly6G+ cells from the lung and blood are graphed in (C). (D-E) Representative flow cytometry plots depicting an alternative gating strategy to confirm neutrophil depletion. Ly6C− CD11b+ C5aR+ SSC-hi cells are primarily neutrophils as indicated by Ly6G expression seen in isotype treated mice (D). This population is significantly reduced in the lungs of anti-Ly6G antibody-treated mice (E). (F) FGF9-OE and control mice were given DOX on d-3, treated with 0.25 mg anti-Ly6G antibody i.p. on d0, infected with 6 x 10^4^ PFU IAV A/WSN/33 i.n. on d0, and monitored for survival. (TIF) [file ppat.1010228.s005.tif]

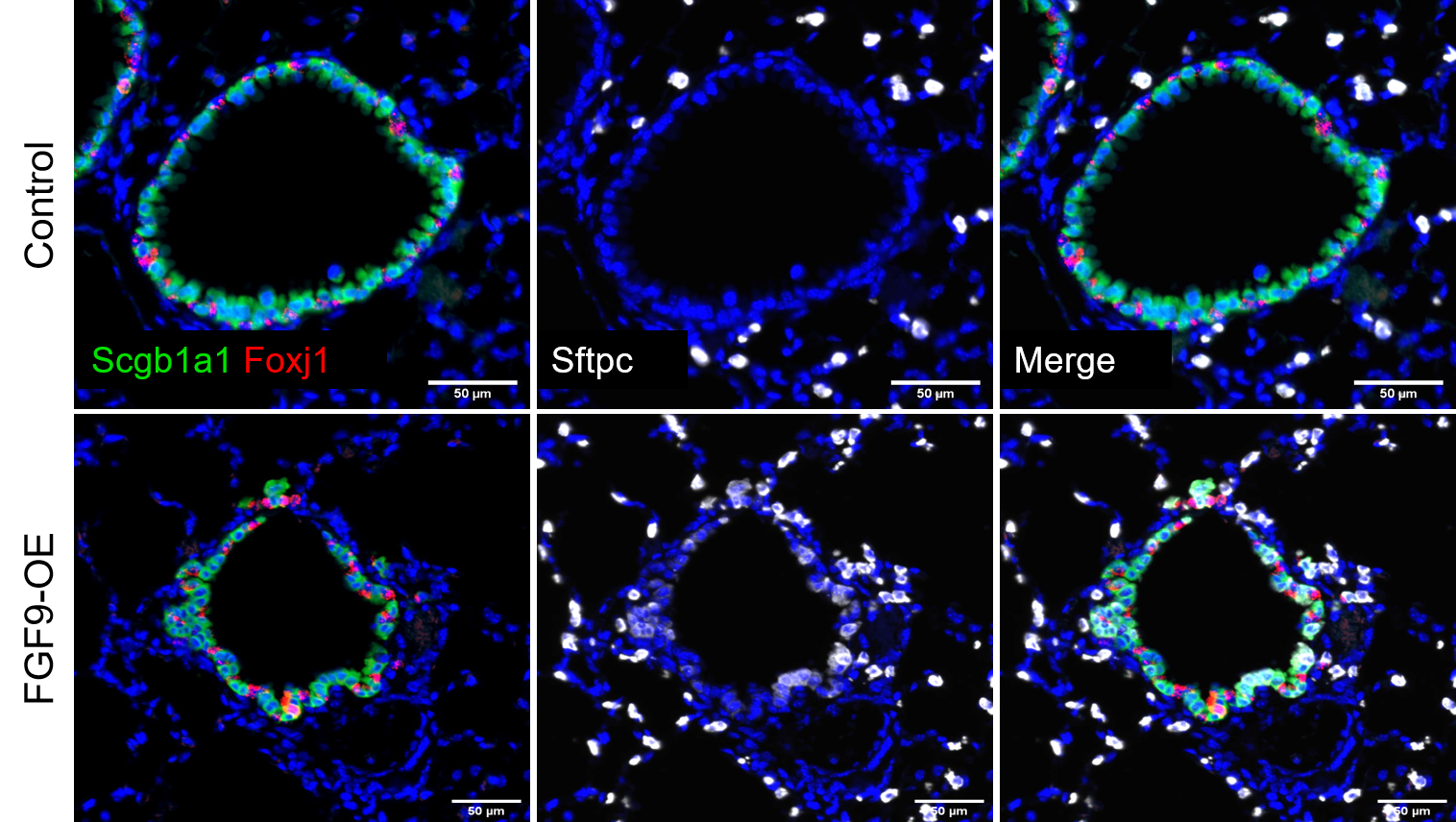

Supplement: S6 Fig — FGF9-OE and control mice were administered DOX beginning on d-3 and lungs were harvested on d0. Shown are representative images of multiplex fluorescent RNA-ISH showing club cells (Scgb1a1, green), AT2 cells (Sftpc, white), and ciliated cells (Foxj1, red) (blue = DAPI). Images taken with 20X objective (scale bars = 50μm). (TIF) [file ppat.1010228.s006.tif]

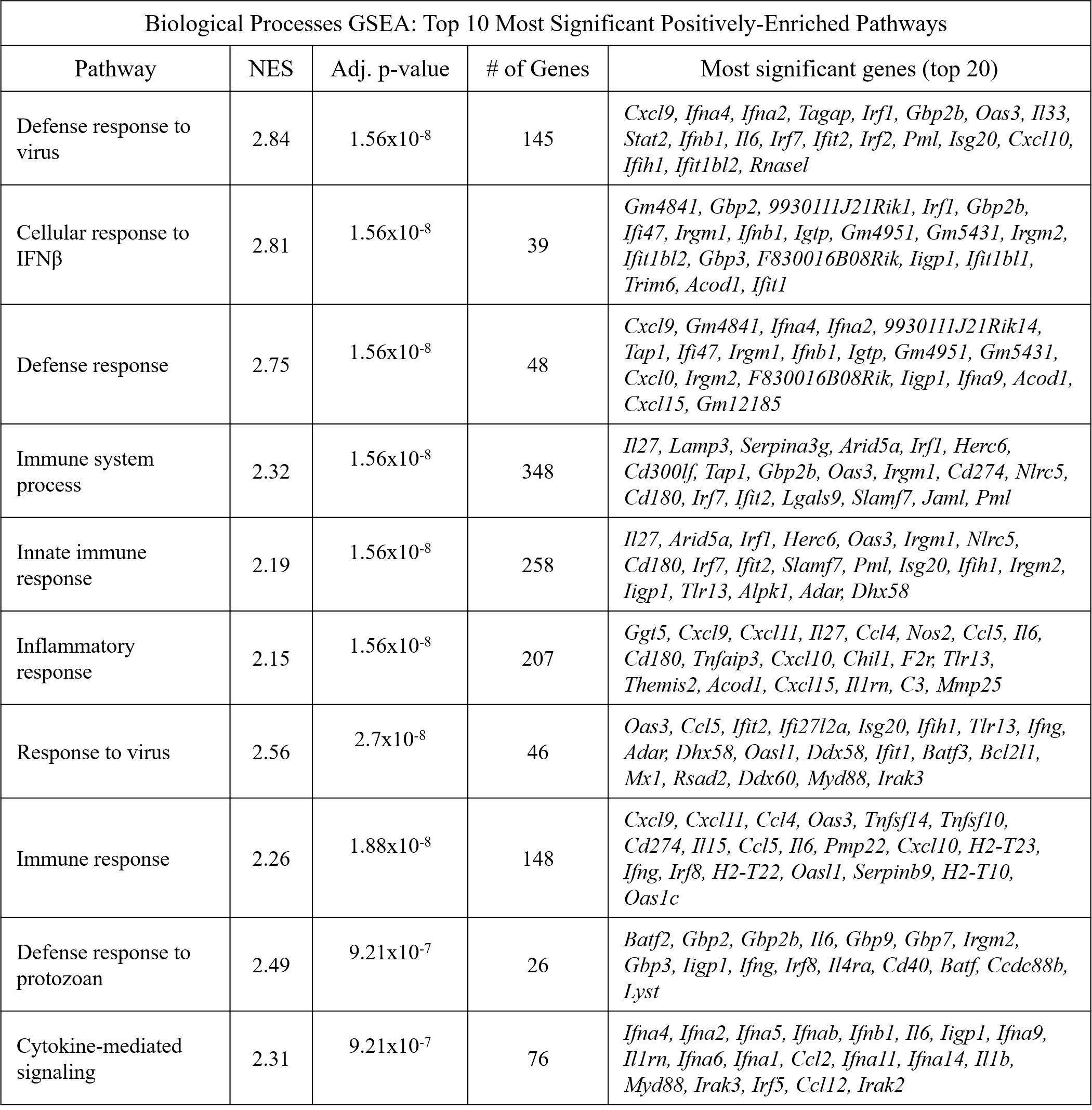

Supplement: S1 Table — Normalized enrichment scores (NES), number of genes, and the top 20 most significant genes in each pathway of the top ten most significant (by adjusted p-value) positively-enriched pathways upregulated in the FGF9-OE airway epithelial cells compared to control airway epithelial cells at 1 dpi. (TIF) [file ppat.1010228.s007.tif]
